# Error in Ninth Paragraph

**DOI:** 10.1001/jamanetworkopen.2019.11076

**Published:** 2019-08-28

The Invited Commentary titled “Medicaid Work Requirements in Kentucky: Identifying the Able-bodied and Holding Them Accountable,”^1^ published July 17, 2019, contained an incorrect statement regarding Michigan’s plan to exempt people living in counties where the unemployment rate exceeds 8.5% from the work requirement. Although an earlier version of Michigan’s waiver included the exemption for counties with high unemployment rates, the final one does not.^2^ This article has been corrected.^1^
